# Astaxanthin Alleviates Autoimmune Hepatitis by Modulating CD8^+^ T Cells: Insights From Mass Cytometry and Single‐Cell RNA Sequencing Analyses

**DOI:** 10.1002/advs.202403148

**Published:** 2024-06-14

**Authors:** Yuting He, Mingjie Ding, Jiakai Zhang, Changjun Huang, Jihua Shi, Yun Wang, Ruolin Tao, Zeyu Wu, Wenzhi Guo

**Affiliations:** ^1^ Department of Hepatobiliary and Pancreatic Surgery The First Affiliated Hospital of Zhengzhou University Zhengzhou 450052 China; ^2^ Key Laboratory of Hepatobiliary and Pancreatic Surgery and Digestive Organ Transplantation of Henan Province The First Affiliated Hospital of Zhengzhou University Zhengzhou 450052 China; ^3^ Open and Key Laboratory of Hepatobiliary & Pancreatic Surgery and Digestive Organ Transplantation at Henan Universities Zhengzhou 450052 China; ^4^ Henan Key Laboratory of Digestive Organ Transplantation Zhengzhou 450052 China

**Keywords:** Astaxanthin, autoimmune hepatitis, CD8^+^ T cell, cytokine, immune microenvironment

## Abstract

Astaxanthin (ASX) is an oxygen‐containing non‐vitamin A carotenoid pigment. However, the role of ASX in autoimmune hepatitis (AIH) remains unclear. In this study, a mouse model of AIH is established induced by concanavalin A (ConA). Mass cytometry and single‐cell RNA sequencing (scRNA‐seq) are used to analyze the potential role of ASX in regulating the immune microenvironment of AIH. ASX treatment effectively alleviated liver damage induced by ConA and downregulated pro‐inflammatory cytokines production in mice. Mass cytometry and scRNA‐seq analyses revealed a significant increase in the number of CD8^+^ T cells following ASX treatment. Functional markers of CD8^+^ T cells, such as CD69, MHC II, and PD‐1, are significantly downregulated. Additionally, specific CD8^+^ T cell subclusters (subclusters 4, 13, 24, and 27) are identified, each displaying distinct changes in marker gene expression after ASX treatment. This finding suggests a modulation of CD8^+^ T cell function by ASX. Finally, the key transcription factors for four subclusters of CD8^+^ T cells are predicted and constructed a cell‐to‐cell communication network based on receptor‐ligand interactions probability. In conclusion, ASX holds the potential to ameliorate liver damage by regulating the number and function of CD8^+^ T cells.

## Graphical abstract

This study revealed that astaxanthin (ASX) treatment effectively alleviated autoimmune hepatitis (AIH) in mice induced by concanavalin A (ConA). Through mass cytometry and single‐cell RNA sequencing, this study deciphered the heterogeneous CD8^+^ T cells profile of AIH liver immune microenvironment regulated by ASX, providing novel insights for the management of AIH.

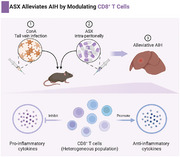

## 1 Introduction

Autoimmune hepatitis (AIH) is a liver disease affecting hepatic parenchyma, which is typically characterized by a T cell‐mediated autoimmune response in genetically susceptible individuals.^[^ ^1^, ^2^, ^3^ ^]^ Particularly, T cells directed against self‐proteins become primed and undergo expansion, thereby initiating and perpetuating liver injury mediated through autoimmune mechanisms.^[^ ^4^ ^]^ According to the guidelines published in 2019, the determination of AIH relies on histological aberrations, distinct clinical, and laboratory indices.^[^ ^5^, ^6^, ^7^ ^]^ Employing steroids in combination with azathioprine as the conventional standard therapeutic approach, has demonstrated significant efficacy in the majority of patients. However, this regimen may cause significant adverse effects, and many patients need to prolong the course of treatment to achieve optimal efficacy. Furthermore, ≈20–30% of individuals cannot achieve full remission despite undergoing this standard treatment protocol.^[^ ^8^ ^]^ Thus, investigating the pathogenesis and exploring novel treatments for AIH are crucial.

The histological hallmark of AIH is periportal or periseptal interface hepatitis, which is characterized by infiltration of macrophages, plasma cells, and lymphocytes.^[^ ^9^, ^10^, ^11^ ^]^ T cells play a predominant role in the immune pathogenesis of AIH.^[^ ^12^ ^]^ Emerging research indicates that in addition to CD4^+^ T cells, CD8^+^ T cells also can play an important role in the disease progression of AIH.^[^ ^13^, ^14^, ^15^ ^]^ However, a comprehensive understanding of the role of CD8^+^ T cells in the pathogenesis of AIH is currently lacking.

Astaxanthin (ASX), a carotenoid pigment naturally synthesized by algae, bacteria, or phytoplankton, is characterized by conjugated double bonds, hydroxyl groups, and ketone groups that participate in electron transfer and quench free radicals.^[^ ^16^, ^17^, ^18^ ^]^ Recent studies have demonstrated the diverse biological activities of ASX, including antioxidant and antineoplastic properties.^[^ ^19^, ^20^, ^21^ ^]^ ASX can inhibit inflammatory cytokines, specifically reduce cyclooxygenase and TNF‐α levels by inhibiting of inducible nitric oxide synthase activity, thus confer a protective effect against hepatitis.^[^ ^22^, ^23^ ^]^ Additionally, ASX potentially elicits immune‐modulatory effects by increasing the production of immunoglobulins and enhancing the response of natural killer (NK) cells and T lymphocytes.^[^ ^24^ ^]^ Previous investigation have found that ASX can play protective effects against renal fibrosis by increasing the population of CD8^+^ T cells, because ASX can upregulate the expression of C‐C chemokine ligand 5 (CCL5) in macrophages.^[^ ^25^ ^]^ Nevertheless, the precise mechanism by which ASX exerts its effects on AIH remains elusive and warrants further research.

In this study, liver injury induced by concanavalin A (ConA) via intravenous injection in mice was used as the animal model to simulate AIH. This is because that ConA‐induced liver damage is primarily caused by T cell activation and recruitment to the hepatic region, which bears significant resemblances to human AIH.^[^ ^26^ ^]^ We intended to evaluate the role of ASX in AIH, and analyze its mechanism by mass cytometry and single‐cell RNA sequencing (scRNA‐seq) to further explore the role of ASX in the progression AIH by regulating CD8^+^ T cells. Our goal was to elucidate the specific mechanisms of ASX to improve AIH, which is anticipated to provide novel perspectives and more reference for the management and prognostication of AIH.

## 2 Results

### 2.1 ASX Attenuates ConA‐Induced AIH in Mice

**Figure 1:**
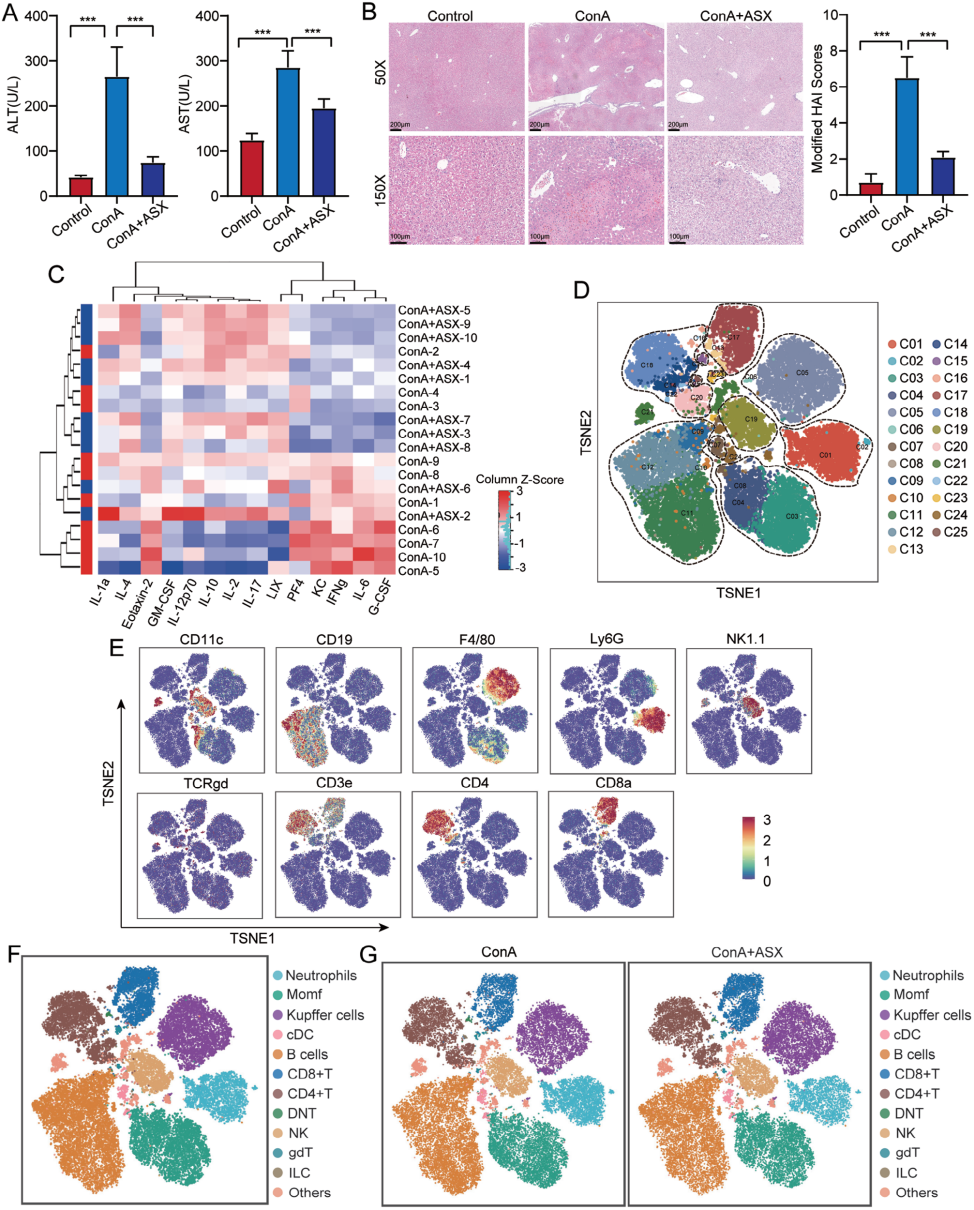
Overview of the alleviating effect of ASX in AIH. A) The levels of ALT and AST. B) Representative images of H&E staining and modified histological activity index (HAI) scores. C) Heatmap displays the expression of cytokines. D) tSNE plots of 25 immune cell subclusters. E) tSNE plot of the representative markers. F) tSNE plots presenting major immune cell types from all groups. (G) tSNE plots presenting major immune cell types in ConA and ConA+ASX groups. ***p < 0.001.

To investigate the role of ASX in the occurrence and development of AIH, we established a ConA‐induced AIH mouse model. We found that ASX treatment significantly alleviated liver damage in mice. The levels of ALT and AST in the ConA+ASX group were significantly lower than those in the ConA group (**Figure** **1**A). Hematoxylin and eosin (H&E) staining revealed massive hepatocyte edema, degeneration, necrosis, and inflammatory cell infiltration in the ConA group, which were alleviated after ASX treatment (Figure 1B). To further explore the potential role of ASX in regulating the inflammatory process, we used the Quantibody Mouse Inflammation Array Kit to comprehensively examine mouse serum, covering 40 cytokines. Compared to the ConA group, we observed a downregulation in pro‐inflammatory factors like KC, IFNg, IL‐6, and G‐CSF in the ConA+ASX group, while the anti‐inflammatory factors IL‐4 and IL‐10 were upregulated (Figure 1C). These results suggested that ASX can alleviate liver injury and inhibit pro‐inflammatory responses in the ConA‐induced AIH mice.

### 2.2 Mass Cytometry Analysis Reveals that ASX May Alleviate Liver Damage by Modulating CD8^+^ T Cells

**Figure 2:**
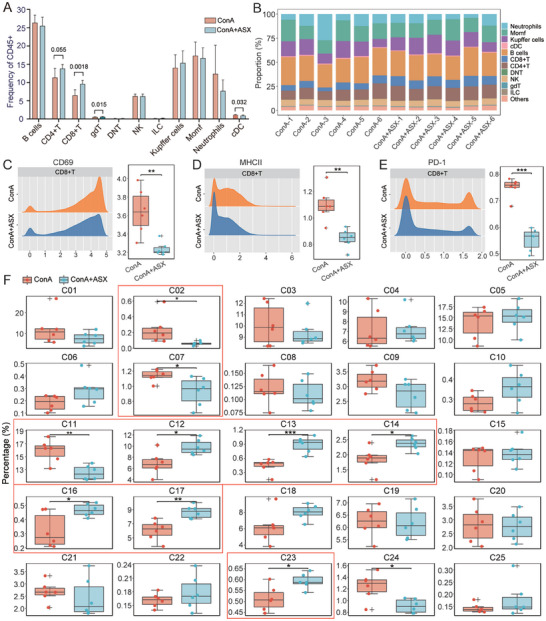
ASX modulation of CD8^+^ T cells in alleviating liver damage based on mass cytometry analysis. A) Differences in the frequency of major immune cell types between the ConA and ConA+ASX groups. B) Distribution of major immune cell types in each sample. C–E) Expression of CD69, MHC II, and PD‐1 between the ConA and ConA+ASX groups. F) Percentage of 25 immune cell subclusters between the ConA and ConA+ASX groups. *p < 0.05, **p < 0.01, and ***p < 0.001.

To further explore the effects of ASX treatment on immune components in the ConA‐induced AIH mouse model, we collected liver tissues from the ConA and ConA+ASX groups for mass cytometry analysis. Immune cells from liver tissues were identified using classical markers, resulting in a total of 25 cell subclusters (Figure 1D; Table S1, Supporting Information). Additionally, we utilized tSNE plots to illustrate the distribution of representative markers (Figure 1E). Next, the immune cells were categorized into eleven major cell types, including neutrophils, monocyte‐derived macrophages (MoMFs), Kupffer cells, conventional dendritic cells (cDCs), B cells, CD8^+^ T cells, CD4^+^ T cells, double‐negative T (DNT) cells, NK cells, gamma delta (gd) T cells, and innate lymphoid cells (ILCs) (Figure 1F,G). Upon comparing the frequency of major cell types between the ConA and ConA+ASX groups, a significant increase of CD8^+^ T cell was observed in the ConA+ASX group (**Figure** **2**A,B). Moreover, the expression of functional markers in CD8^+^ T cells, such as CD69, MHC II, and PD‐1, was significantly downregulated in the ConA+ASX group (Figure 2C–E). Subsequently analyses of the CD8^+^ T cell subclusters aimed to further elucidate the impact of ASX on CD8^+^ T heterogeneity. The results revealed that C13, identified as Ly6C^hi^TCF7^+^TCRb^+^CD127^lo^CD62L^lo^ immune‐active central memory CD8^+^ T cells, and C17, recognized as CD103^+^TCF7^hi^Tbet^+^CD69^hi^TCRb^+^ resident CD8^+^ T cells, were notably increased in proportion in the ConA+ASX group (Figure 2F). These findings suggest that ASX may alleviate liver damage by regulating the number and function of CD8^+^ T cells.

### 2.3 scRNA‐seq Analysis Indicates that ASX Regulates the Liver Immune Profile of ConA‐Induced AIH Mice

**Figure 3:**
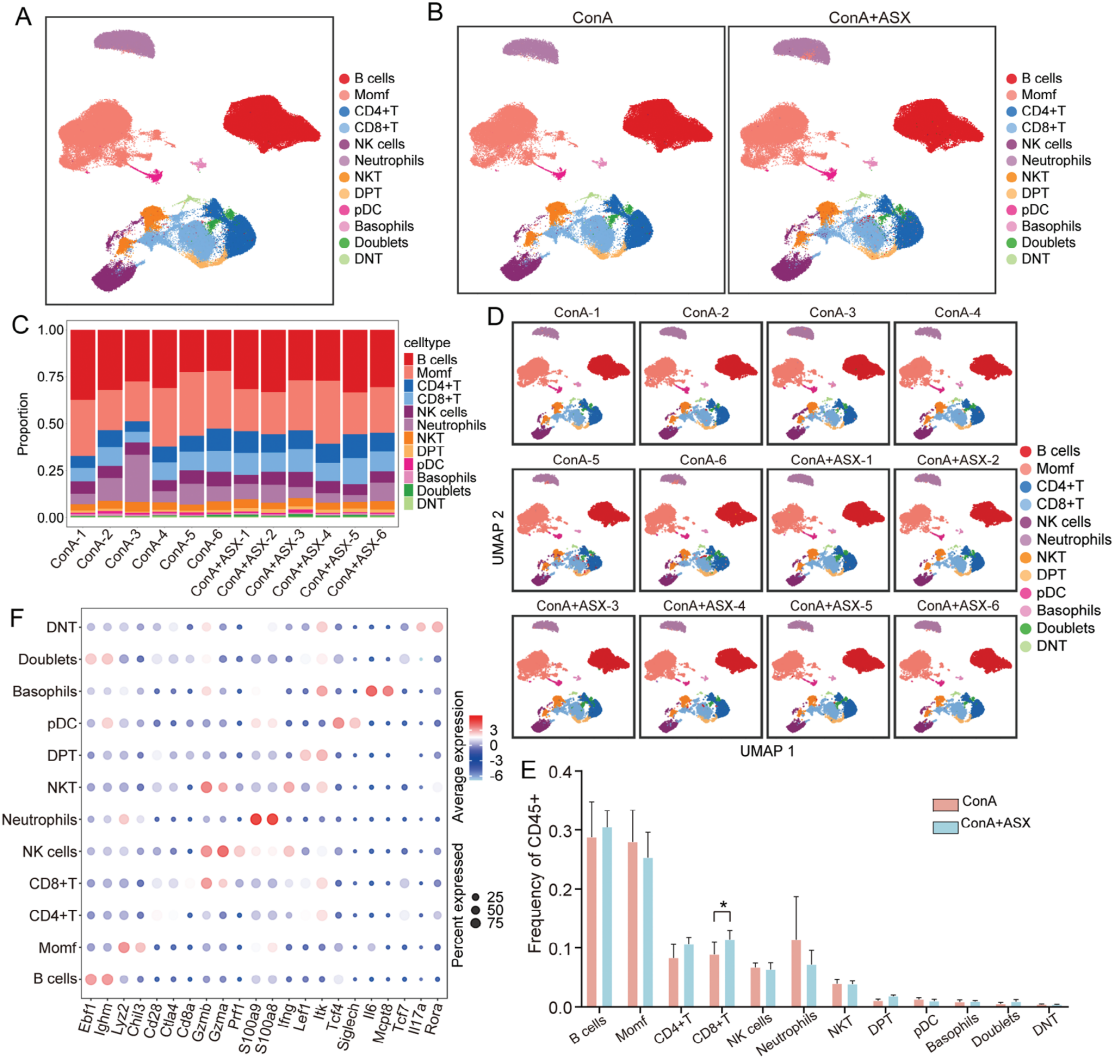
The liver immune profile of ConA‐induced AIH mice based on scRNA‐seq analysis. A) UMAP plot of twelve immune cell populations. B) UMAP plots of twelve immune cell populations in the ConA and ConA+ASX groups. C) Frequencies of immune cell populations in each sample. D) Distribution of immune cell populations in each sample. E) Frequency of immune cell populations between the ConA and ConA+ASX groups. F) Expression of the top two key genes in different immune cell populations. *p < 0.05.

To further explore the role of ASX in mitigating liver injury by modulating immune cells, we collected liver tissues from the ConA and ConA+ASX groups for scRNA‐seq analysis. Through unsupervised cluster analysis, we identified twelve immune cell populations, namely B cells, MoMFs, CD4^+^ T cells, CD8^+^ T cells, NK cells, neutrophils, NKT cells, double‐positive T (DPT) cells, plasmacytoid DCs (pDCs), and basophils, doublets, and DNT cells (**Figure** **3**A,B). Bar plots present the frequency of each immune cell population in each sample (Figure 3C), and uniform manifold approximation and projection (UMAP) plots visualized the quantity and distribution of immune cell population in each sample (Figure 3D). A significant difference was observed in the frequency of CD8^+^ T cells between the ConA and ConA+ASX groups (Figure 3E). This finding was consistent with the results of mass cytometry analysis.

To further explore the immunological mechanism of ASX in improving ConA‐induced liver injury, we analyzed the key gene of immune cell populations (Figure 3F). The results suggested that each immune cell population displayed a particular gene expression pattern. Neutrophils, primarily expressing S100A8 and S100A9, play a pro‐inflammatory, antibacterial, and immunomodulatory role. Basophils were identified as the primary source of IL6 and MCPT8. IL‐6 can promote the migration and activation of basophils, while MCPT8 is involved in particle release and matrix degradation, thereby enhancing the inflammatory response. Granzyme A (GZMA) and GZMB were expressed in NK cells, NKT cell, and CD8^+^ T cells, promoting cell apoptosis through direct interaction with target cells or extracellular release.

### 2.4 ASX Alleviates Liver Damage by Regulating Multiple CD8^+^ T Cell Subclusters

**Figure 4:**
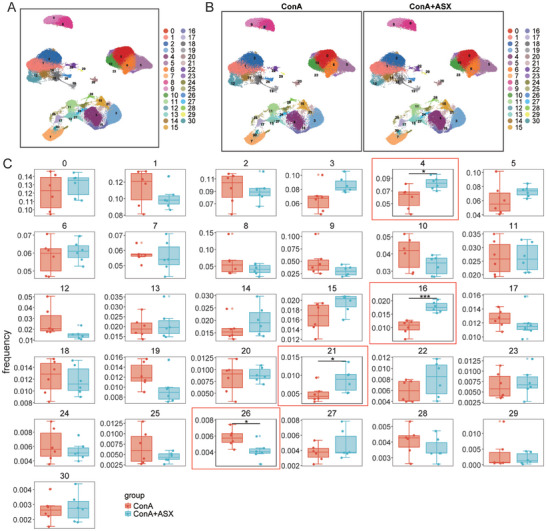
ASX regulates the liver immune profile in ConA‐induced AIH mice. A) Identification of 31 immune cell subclusters through scRNA‐seq analysis. B) Distribution of 31 immune cell subclusters in the ConA and ConA+ASX groups. C) Frequency of different immune cell subclusters between the ConA and ConA+ASX groups. *p < 0.05, ***p < 0.001.

**Figure 5:**
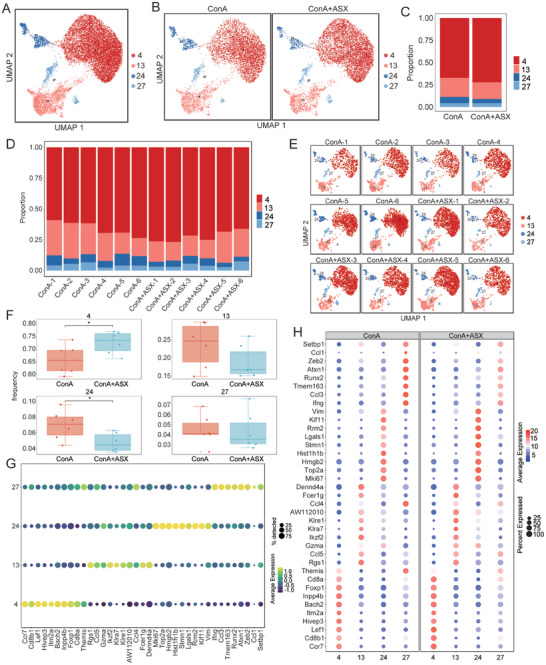
Differences in subclusters 4, 13, 24, and 27 in AIH. A,B) UMAP plots visualize the distribution of subclusters 4, 13, 24, and 27. C) Proportion of subclusters 4, 13, 24, and 27 in the ConA and ConA+ASX groups. D) Proportion of subclusters 4, 13, 24, and 27 in each sample. E) UMAP plots visualize the distribution of subclusters 4, 13, 24, and 27 in each sample. F) Frequencies of subclusters 4, 13, 24, and 27 between the ConA and ConA+ASX groups. G) Top ten key genes of subclusters 4, 13, 24, and 27 in all samples. H) Top ten key genes of subclusters 4, 13, 24, and 27 between the ConA and ConA+ASX groups. *p < 0.05.

We identified 31 immune cell subclusters based on the expression of marker genes (**Figure** **4**A; Table S2, Supporting Information). The distribution of immune cell subclusters in the ConA+ASX group differed from that in the ConA group (Figure 4B). The proportion of subcluster 4, 16, and 21 were significantly upregulated, whereas that of subcluster 26 was significantly downregulated in the ConA+ASX group (Figure 4C). To further explore the effects of ASX treatment on liver CD8^+^ T cells, four subclusters of CD8^+^ T cells were included in the downstream analysis, namely CD3d^+^GZMB^+^LEF1^+^CD8^+^ T cells (subcluster 4), CD3e^+^KLRA7^+^CHN2^+^CCL5^+^TOX^+^CD8^+^ T cells (subcluster 13), CD3d^+^TOP2a^+^MKi67^+^STMN1^+^CD8^+^ T cells (subcluster 24), CD3d^+^NKG7^+^IFNg^+^GZMB^+^CD8^+^ T cells (subcluster 27). The UMAP plots depicted the distribution of subclusters 4, 13, 24, and 27 (**Figure** **5**A,B). In the ConA+ASX group, the proportions of subclusters 4, 13, 24, and 27 differed from those in the ConA group (Figure 5C). The frequencies and distribution of subclusters 4, 13, 24, and 27 in each sample are shown in Figure 5D,E. Subcluster 4 exhibited increased frequencies, whereas subcluster 24 was markedly decreased in the ConA+ASX group (Figure 5F).

We further analyzed the expression of the top ten marker genes in different subclusters (Figure 5G). *Ccr7*, *Cd8b1*, *Lef1*, *Hivep3*, *Itm2a*, *Bach2*, *Inpp4b*, *Foxp1*, *Cd8a*, and *Themis* were the top ten marker genes of subcluster 4. These genes indicated that subcluster 4 was closely related to the early stages of the immune response and the migration within lymph nodes. They may exhibit high MHC‐I recognition, cell proliferation, and survival, playing a crucial role in the development of antigen‐specific T cell responses. The expression of the top ten marker genes in subcluster 4 was significantly upregulated after ASX treatment, suggesting enhanced MHC‐I recognition and cell proliferation (Figure 5H). Subcluster 13 was involved in the migration of immune cells to the inflammation sites by releasing chemokines such as Ccl5 and Ccl4. After ASX treatment, subcluster 13 exhibited a higher ability to release chemokines such as Ccl4 and played its cytotoxic role by encoding GZMA. The top ten marker genes of subcluster 24 were linked to cell cycle, DNA repair, and transcriptional regulation. These genes may play a critical role in promoting T cell proliferation and maintaining antigen‐specific responses, thus potentially contributing to the formation of immunological memory. After ASX treatment, the cytotoxic activity of subcluster 27, responsible for producing interferon, was diminished. The expression levels of the top ten marker genes in different subclusters further validated the distinct roles and regulatory mechanisms of CD8^+^ T cells in the immune response.

**Figure 6:**
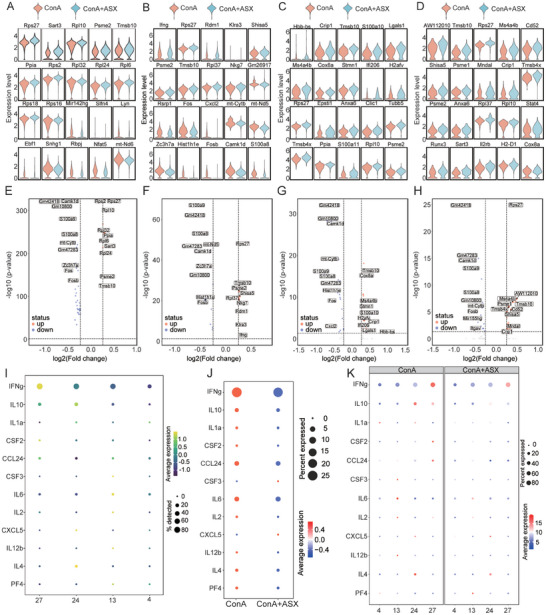
DEGs and cytokine profiles of CD8^+^ T cell subclusters. A–D) Top 20 DEGs expression levels of (A) subcluster 4, (B) subcluster 13, (C) subcluster 24, and (D) subcluster 27 between the ConA and ConA+ASX groups. E–H) Volcano plots of DEGs in (E) subcluster 4, (F) subcluster 13, (G) subcluster 24, and (H) subcluster 27. I) Gene expression of typical cytokines in four CD8^+^ T cell subclusters across all samples. J) Gene expression of typical cytokines between the ConA and ConA+ASX groups. K) Gene expression of typical cytokines in four CD8^+^ T cell subclusters between the ConA and ConA+ASX groups.

To delve into the functions of CD8^+^ T cell subclusters, we examined differential expression genes (DEGs) in subclusters 4, 13, 24, and 27 between the ConA and ConA+ASX groups (**Figure** **6**A–D). Volcano plots illustrate the upregulated and downregulated DEGs between the two groups. ASX treatment led to a significant upregulation in the expression of *Sart3* and *Psme2* in subcluster 4 (Figure 6E). This suggested that the functionality of subcluster 4 was enhanced in terms of immune response and antigen processing. The upregulation of *Tmsb10* may promote the proliferation and differentiation of subcluster 4. Reduced expression of *Fos* and *S100a8* indicated suppressed inflammatory cascade, suggesting a reinforced anti‐inflammatory capacity of subcluster 13 (Figure 6F). The increased expression of *Ms4a4b* in subcluster 24 potentially promoted the activation and proliferation of CD8^+^ T cells, ultimately improving the effectiveness of immune responses (Figure 6G). The upregulation of *Ms4a4b*, *Cd52*, *Psme1*, and *Psme2* may have a positive effect on the immune function of subcluster 27 (Figure 6H), resulting in increased activation and proliferation of subcluster 27, thereby promoting the antigen recognition and presentation process by CD8^+^ T cells.

### 2.5 ASX Regulates the Cytokines in AIH

The differences in cytokine profiles between the ConA and ConA+ASX groups are shown in Figure 1C. We investigated the gene expression of typical cytokines in each CD8^+^ T cell subcluster and identified which CD8^+^ T cell subclusters drive these cytokines. Although IFNg was found to be expressed in all four CD8^+^ T cell subclusters, it was primarily expressed in subcluster 27 (Figure 6I). IL‐4 was highly expressed in subcluster 24, whereas IL‐6 and IL‐2 were predominantly expressed in subcluster 13. Although these 12 cytokines were expressed in subcluster 4 of CD8^+^ T cells, their expression levels were relatively low. Overall, the expression levels of cytokines such as IFNg, IL‐6, IL‐2, and IL‐4 were reduced following ASX treatment (Figure 6J). The marked decrease in the cytokine‐secreting capacity of CD8^+^ T cells suggests functional inhibition caused by ASX. Further detailed analysis revealed that the decrease in IFNg expression in the ConA+ASX groups was primarily attributed to a reduction in IFNg expression in subcluster 27, and the reduction in IL‐4 levels in the ConA+ASX groups was due to the downregulation of IL‐4 in subcluster 24 (Figure 6K).

### 2.6 Pseudo‐Time Analysis Revealed the Differentiation Stages of CD8^+^ T Cells

**Figure 7:**
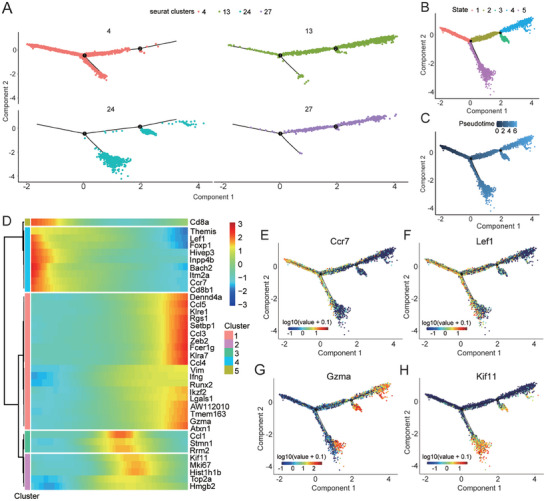
CD8^+^ T cell differentiation trajectory. A,B) Subclusters 4, 13, 24, and 27 were categorized into five differentiation states. C) The developmental trajectory of CD8^+^ T cell subclusters over time. D) Gene expression along the pseudo‐time progression. E–H) Gene expression of (E) *Ccr7*, (F) *Lef1*, (G) *Gzma*, and (H) *Kif11* along the pseudo‐time trajectory.

To explore the developmental stages of CD8^+^ T cell subclusters, we utilized Monocle 2,^[^ ^27^ ^]^ an algorithm based on transcriptomic similarity, to pseudo‐time reorder the cells and reconstruct the lineage of biological processes. Subclusters 4, 13, 24, and 27 can be broadly classified into five differentiation states (**Figure** **7**A,B). The developmental stage of CD8^+^ T cell subclusters over time is depicted in Figure 7C, where lighter colors represent later stages of development. We observed that subcluster 4 was mainly in the initial stage of development and may potentially differentiate into subclusters 13, 24, and 27. Additionally, we observed distinct changes in gene expression along the pseudo‐time progression and identified five major clusters of transcriptional gene modules (Figure 7D). *Ccr7* and *Lef1*, indicating naive immune characteristics, showed as gradual decrease in expression along the pseudo‐time path. (Figure 7E,F). The expression levels of *Gzma* and *Klre1*, associated with the function and cytotoxicity of CD8^+^ T cells, gradually increased along the pseudo‐time path (Figure 7G,H).

### 2.7 Construction of a Transcription Factor Regulatory Network for CD8^+^ T Cells in AIH

Transcription factors establish regulatory relationships with target genes by binding to specific DNA sequences, thereby regulating gene expression. To deepen our understanding of the regulatory mechanisms governing marker gene expression in immune cells, we conducted network analysis to predict key transcription factors across four distinct subclusters of CD8^+^ T cells (Figures S1–S4, Supporting Information). The central transcription factors identified in subcluster 4 were Tfdp1, Foxp1, Bach2, Hdac2, and Myc (Figure S5A, Supporting Information). Hdac2 primarily regulates chromatin structure and gene transcriptional activity. Tfdp1 was a member of the E2F transcription factor family involved in the regulation of the cell cycle. Tfdp1 formed complexes with other members of the E2F family and participated in the transcriptional activation of target genes in subcluster 4, thus regulating cell cycle progression. The central transcription factors identified in subcluster 13 were Hhex, Runx1, Arid3a, Hnf1a, and Ebf1 (Figure S5B, Supporting Information). Hhex and Runx1 were observed to play critical roles in the development and differentiation of subcluster 13. The loss of Hhex or Runx1 can lead to developmental disorders in subcluster 13, affecting the generation and function of subcluster 13. Foxp3 was found to play a central role as a transcription factor in regulating subclusters 24 and 27 in AIH (Figure S5C,D, Supporting Information). Foxp3 was critically involved in regulating the function and development of subclusters 24 and 27. It imparted immunosuppressive capabilities to subclusters 24 and 27, contributing to the maintenance of immune balance and self‐tolerance.

### 2.8 Developing an Interaction Network of CD8^+^ T Cell Subclusters to Uncover their Involvement in AIH

**Figure 8:**
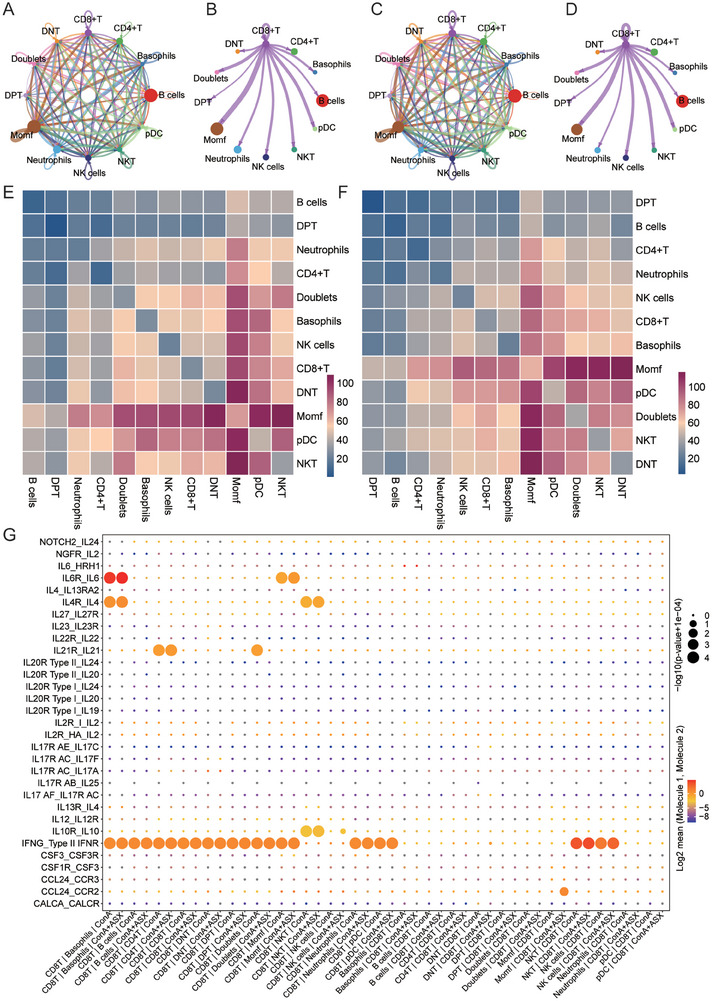
Interactions among CD8^+^ T cell subclusters. A) Interactions among multiple immune cell types in the ConA group. B) Interactions between CD8^+^ T cells and other immune cell types in the ConA group. C) Interactions among multiple immune cell types in the ConA+ASX group. D) Interactions between CD8^+^ T cells and other immune cell types in the ConA+ASX group. E) Heatmap displayed the strength of cell–cell interactions in the ConA group. F) Heatmap displayed the strength of cell–cell interactions in the ConA+ASX group. G) Ligand–receptor pairs between CD8^+^ T cells and multiple immune cell types.

AIH is a liver disorder characterized by aberrant immune system activity. Various immune cell contributes significantly to its pathogenesis and progression. To characterize the complex communication between different immune cell types in the liver immune microenvironment of AIH, we employed cellphoneDB (v2) to construct a cell–cell interaction network.^[^ ^28^ ^]^ Diverse interactions were observed between multiple immune cell types, including MoMFs, B cells, CD4^+^ T cells, and CD8^+^ T cells (**Figure** **8**A–D). CD8^+^ T cells demonstrated a greater possibility to interact with various immune cell types, including MoMFs, pDCs, and NKT cells, with MoMFs accounting for most of the interactions (Figure 8E). After ASX treatment, the interaction between CD8^+^ T cells and MoMFs was relatively weakened (Figure 8F). CD8^+^ T cells and MoMFs can communicate with each other by ligand‐receptor pairs such as IFNg and Type II IFNR as well as IL6 and IL6R (Figure 8G). ASX treatment led to an increased CCL24‐CCR2 interaction between CD8^+^ T and MoMFs. Besides, the communication between CD8^+^ T and NK cells might occur through interaction involving IL10 and IL10R. Taken together, these findings indicated that CD8^+^ T cells may communicate with liver immune cell through complicated ligand‐receptor interaction network.

## 3 Discussion

ASX, a carotenoid pigment derived from oxygen‐containing non‐vitamin A sources, plays a role in relieving various disease progression by its anti‐aging, anti‐inflammatory, and anti‐apoptotic activities.^[^ ^29^ ^]^ In CCL4‐administered rats, ASX alleviated liver fibrosis by inhibiting lipid peroxidation and enhancing the cellular antioxidant system.^[^ ^30^ ^]^ In non‐alcoholic fatty liver disease (NAFLD), ASX exhibited inhibitory effects on weight gain and adipose tissue increase in obese mice fed a high‐fat diet.^[^ ^31^ ^]^ In liver cancer, ASX inhibited cell proliferation and promoted cell apoptosis by interacting with key molecules in signaling pathways.^[^ ^32^, ^33^ ^]^ Moreover, ASX alleviated drug‐induced liver injury by reducing the level of inflammatory factors.^[^ ^34^, ^35^ ^]^ AIH is a disease characterized by liver tissue damage due to abnormal immune system reactions. The research on the role of ASX in AIH is limited.

To study the effect of ASX in AIH, we employed the ConA‐induced AIH mouse model. We found that ASX treatment significantly alleviated liver damage in ConA‐induced mice compared with that in the control group. Immune dysregulation is a fundamental mechanism underlying AIH pathogenesis. Changes in immune cell profiles in AIH have been documented in multiple studies.^[^ ^36^, ^37^, ^38^ ^]^ However, the specific roles and mechanisms of these immune cells in AIH remain to be elucidated. Our findings indicated that ASX treatment led to alterations in multiple pro‐inflammatory factors in the ConA+ASX group. Therefore, we speculated that ASX may alleviate liver injury by regulating cytokine secretion in AIH. Mass cytometry and scRNA‐seq revealed a significant increase in the number of CD8^+^ T cells after ASX treatment. The expression of functional markers CD69, MHC II, and PD‐1 in CD8^+^ T cells was significantly downregulated, indicating altered activation status and regulatory function of CD8^+^ T cells. The involvement of CD8^+^ T cells in emperipolesis has been observed in patients with AIH, potentially leading to the apoptosis of both CD8^+^ T cells and hepatocytes.^[^ ^39^ ^]^ Additionally, Ren and colleagues reported that ASX promoted the infiltration of CD8^+^ T cells in the HCC tumor microenvironment by activating CXCL9/CXCR3 signaling axis, thereby enhancing the body's anti‐tumor immune response.^[^ ^40^ ^]^ The discovery of the role of ASX in regulating CD8^+^ T cells in AIH is novel and deserves further exploration.

To gain a deeper insight into the mechanism through which ASX alleviates liver injury, we assessed the expression of functional genes within CD8^+^ T cell subclusters and analyzed their potential roles. Distinct CD8^+^ T cell subclusters exhibited significant differential expression after ASX treatment, indicating a functional alteration in CD8^+^ T cells following ASX treatment. While we elaborated on the roles of differentially expressed markers through scRNA‐seq, further cell experiments are needed to comprehensively clarify the functions of these markers. Additionally, we observed significant differences in plasma inflammatory factors between the ConA and ConA+ASX groups in mice. Using scRNA‐seq, we investigated the expression of cytokines after ASX treatment and identified the cell sources of these differential cytokines. These findings contribute to advancing our understanding of the mechanism by which ASX alleviates AIH. Transcription factors play a crucial role in regulating the differential expression of marker genes. Through network analysis, we predicted key transcription factors in four distinct CD8^+^ T cell subclusters. The interactions among various subtypes of immune cells are implicated in the progression of AIH. We constructed a cell–cell communication network by calculating communication probabilities, demonstrating the interactions among immune cell subtypes and presenting their patterns. The comparison of the cell–cell communication network before and after ASX treatment has enhanced our understanding of the role of immune cell interactions in the progression of AIH.

To our knowledge, this study represents the first to combine mass cytometry and scRNA‐seq to investigate the impact of ASX administration on the liver single‐cell immune landscape in AIH mice. Although our study indicated the regulatory effect of ASX on different CD8^+^ T cells subpopulations in the AIH liver immune microenvironment, additional mechanism research in vivo and in vitro is necessary to further validate this finding. For example, liver‐resident primary CD8^+^ T cells can be isolated by flow cytometry sorting to explore the direct regulatory effects of ASX on CD8^+^ T cells. Additionally, liver organoid model derived from human and mouse liver could be established to enhance our understanding of the effect of ASX.

In conclusion, our findings suggest that ASX may alleviate liver injury by regulating the number and function of CD8^+^ T cells. Additionally, we elucidated changes in the immune microenvironment of AIH after ASX treatment, and proposed ASX as a novel therapeutic strategy for restoring immune function in AIH.

## 4 Experimental Section

### Reagents

ConA was obtained from Biosharp (Beijing, China), and ASX was acquired from Energy Chemical (Shanghai, China). In total, 42 antibodies utilized for CyTOF analysis were sourced from BioLegend (San Diego, USA), eBioscience (San Diego, USA), BD Biosciences (San Jose, USA), Bio‐Rad (Hercules, USA), and R&D Systems (Minneapolis, USA). Detailed antibody information is provided in Table S3 (Supporting Information).

### Animal Experiments

C57BL/6 mice were procured from Vital River Laboratory Animal Technology (Beijing, China). The mice were housed under controlled conditions (temperature: 22 °C, humidity: 55%, 12 h light/dark cycle). The experiments were performed following the National Institutes of Health Guide for the Care and Use of Laboratory Animals and were approved by the ethical guidelines of the Zhengzhou University School of Medicine Experimental Animal Management and Ethics Committee (ZZU‐LAC20231201‐241).

C57BL/6 mice were randomized to three groups: ConA+ASX group (*n* = 10), ConA group (*n* = 10), and control group (*n* = 10). Mice underwent an 8 h fast with unrestricted water intake before the experiment. ASX was dissolved in olive oil at 40 mg mL^−1^ and ConA were dissolved in normal saline. Mice in the ConA+ASX group were intraperitoneally injected with ASX at 200 mg kg, whereas the control group received olive oil. Subsequently, ConA was administered to mice in both the ConA+ASX and ConA groups via tail vein injection at 15 mg kg^−1^. Mice were then switched to normal chow following ConA treatment. Twelve hours after establishing the ConA mouse model, blood and liver samples of mice were collected following anesthesia.

### Histological Analyses

H&E staining was performed to evaluate the extent of liver injury. According to previously reported protocols,^[^ ^41^, ^42^ ^]^ the liver tissue was fixed in 4% paraformaldehyde for 24 h, dehydrated using 50% ethanol, embedded in paraffin, and sectioned. Liver slides were finally stained with HE, dehydrated, and sealed.

### Cytokine Array

A Quantibody Array glass chip (RayBiotech, Inc., Guangzhou, China) was used to assess the relative expression levels of 40 mouse cytokines. The serum was diluted and incubated on a glass chip array according to the manufacturer's protocol. Images were obtained using the FluorChem M system (ProteinSimple, USA), and protein expression levels were quantified using ImageJ software.

### Single‐Cell RNA Sequencing

Following the manufacturer's recommendations, scRNA‐seq was performed using the MoboNova‐100 Genomics platform (ConA group vs ConA+ASX group, *n* = 6 vs *n* = 6). In detail, cDNA from different cell origins was pooled, PCR‐amplified for quality inspection, and the resulting libraries were sequenced. Reads with low quality, contaminated joints, and a high content of unknown base N were excluded. Data analysis was conducted using the Cell Ranger 2.0.0 software, which facilitated the management and analysis of sequencing data. This included mapping reads to the reference genome, quantifying gene expression levels, and detecting transcriptional events. Further data processing and cluster analysis were carried out using the Seurat R package, which enabled the identification of distinct cellular clusters and transcriptional profiles. For the visualization and further exploration of the data, dimensionality reduction was performed using the UMAP algorithm.

### Mass Cytometry

Single‐cell suspensions were prepared from harvested liver tissues (ConA group vs ConA+ASX group, *n* = 6 vs *n* = 6). Each sample was subjected to a standardized protocol involving thorough cleaning to remove debris and non‐parenchymal cells, enzymatic digestion using a collagenase‐hyaluronidase blend to dissociate cells, and centrifugation at 300 g for 5 min to pellet the cells. To differentiate live from dead cells, a stain with isotopically pure 194Pt was used, due to its effective binding to cellular proteins in dead cells while sparing live cells. After washing, cells were incubated with Fc receptor‐blocking agents to minimize non‐specific antibody binding. For extracellular staining, cells were exposed to a cocktail of metal‐labeled antibodies targeting specific cell surface markers. Following extracellular labeling, cells were fixed using a paraformaldehyde solution to preserve cell morphology and prevent degradation. Intracellular staining was then performed to detect cytokines and other intracellular proteins, crucial for understanding cellular function and state. Stained samples were analyzed using a Helios mass cytometer, operated as per the manufacturer's guidelines. Raw data files were preprocessed to correct for any instrumental artifacts and normalize signal intensity. Data were then subjected to clustering algorithms to identify distinct cell populations, followed by visualization in multidimensional plots to illustrate the immune landscape of the samples.

### Statistical Analysis

Quantitative data were presented as mean ± standard deviation. Student's unpaired t‐test was used to determine significant differences between the two groups. Statistical significance was set at two‐sided *p* < 0.05.

## Conflict of Interest

The authors declare no conflict of interest.

## Author Contributions

Y.H., M.D., and J.Z. contributed equally to this work. Y.H. and W.G. conceptualized and supervised this study. Y.H., M.D., and J.Z. participated in the overall experiments and wrote the draft. C.H., J.S., and Y.W. collected and analyzed data. R.T. and Z.W. created the figures and tables. All authors read and approved the final manuscript.

## Supporting information

Supporting Information

## Data Availability

The data that support the findings of this study are available from the corresponding author upon reasonable request.
