# Supplementary material for: Astaxanthin Alleviates Autoimmune Hepatitis by Modulating CD8+ T Cells: Insights From Mass Cytometry and Single‐Cell RNA Sequencing Analyses
Source: Adv Sci (Weinh). 2024 Jun 14;11(30):2403148. doi: 10.1002/advs.202403148 (PMC11321693; doi:10.1002/advs.202403148)
Supplement: Supplementary file 1 — Supporting Information [file ADVS-11-2403148-s001.docx]

Supplementary Materials for

**Astaxanthin alleviates Autoimmune Hepatitis by Modulating CD8^+^ T Cells: Insights from Mass Cytometry and Single-Cell RNA Sequencing analyses**

Yuting He *et al.*

**Corresponding authors:** Yuting He, fccheyt1@zzu.edu.cn; Wenzhi Guo, fccguowz@zzu.edu.cn

**The supplementary file includes:**

**Figure S1 to Figure S5**

**Table S1 to Table S3**

**
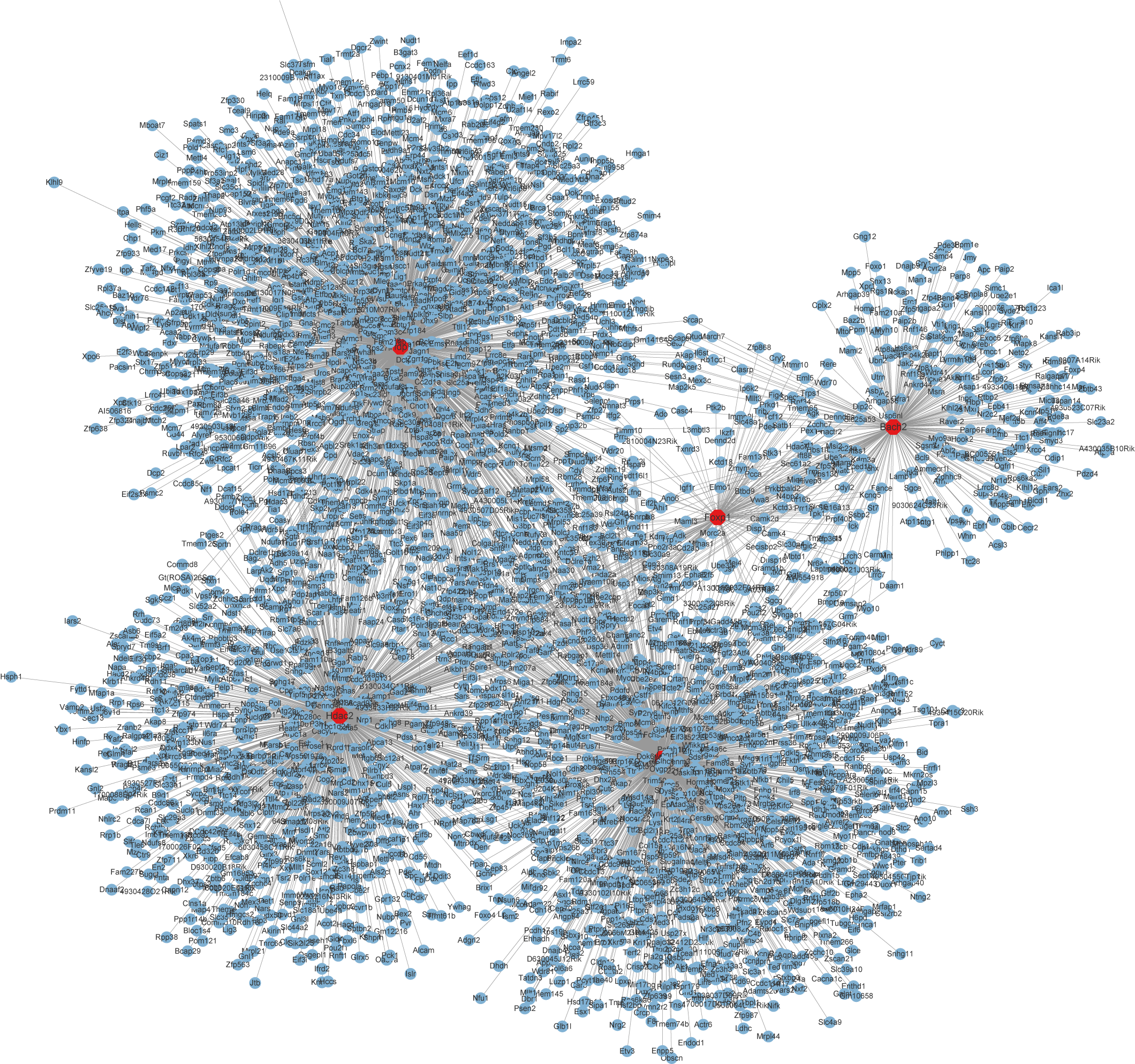
**

**Figure S1. Transcription factor regulatory network for subclusters 4.**


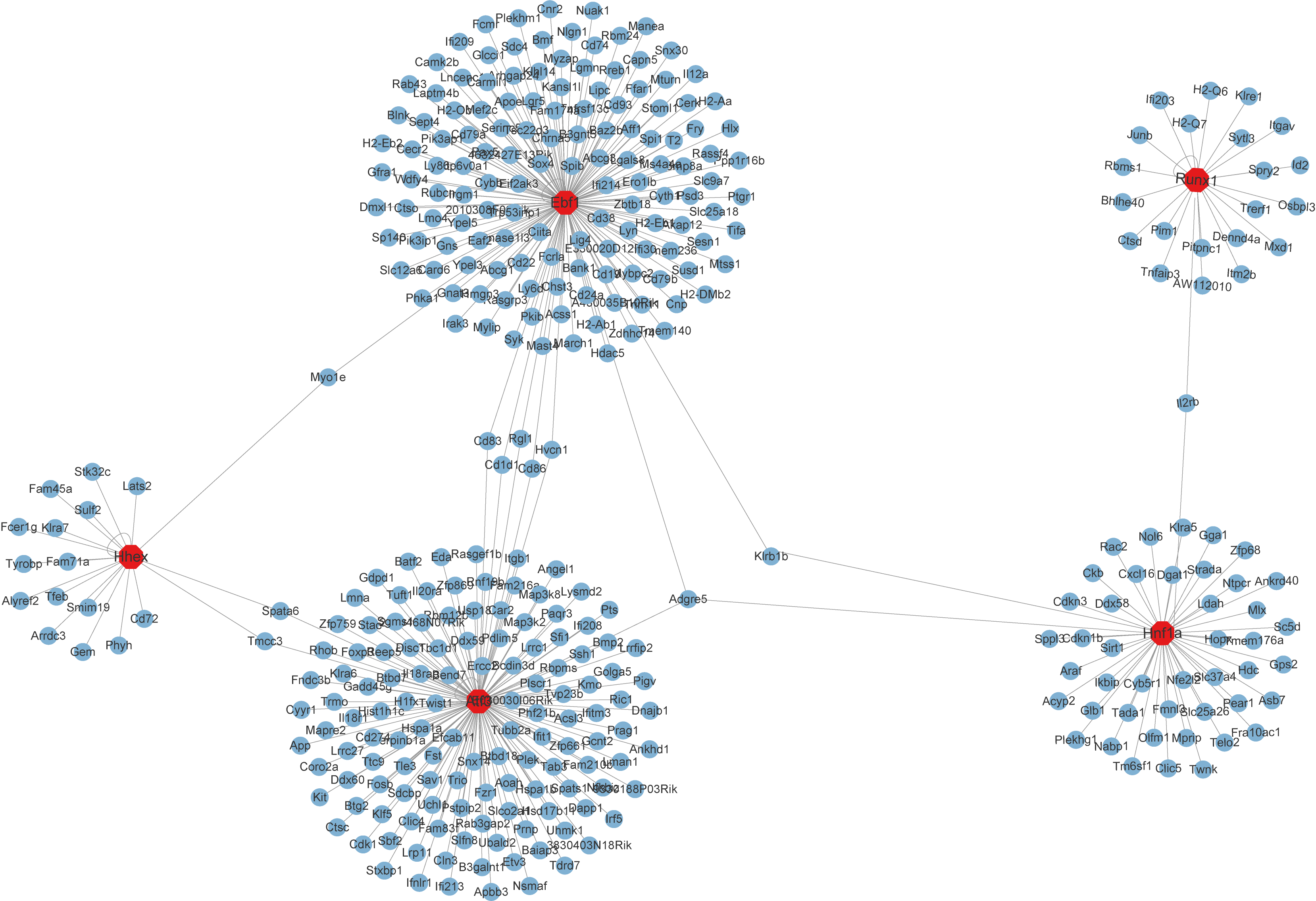


**Figure S2. Transcription factor regulatory network for subclusters 13.**

**
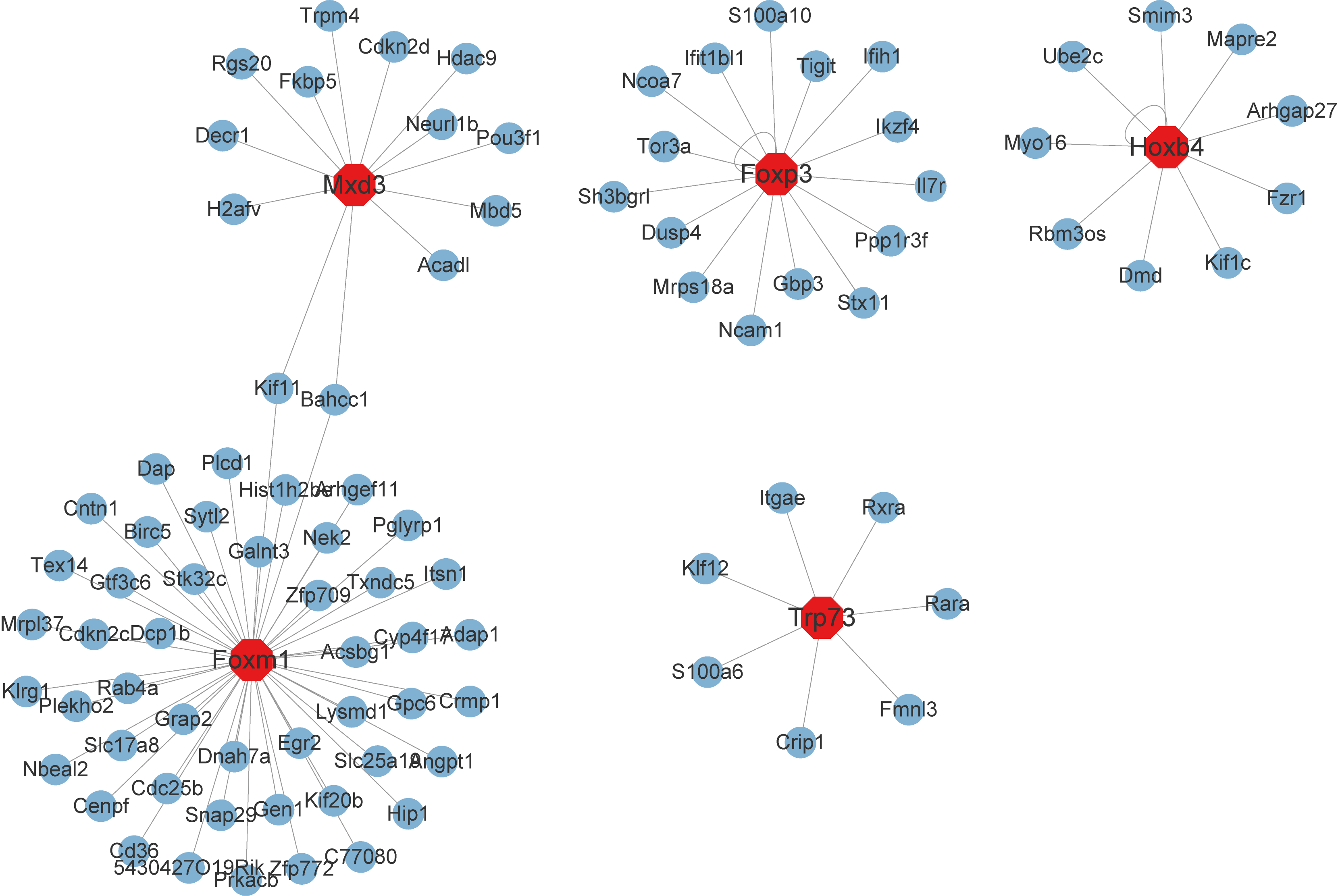
**

**Figure S3. Transcription factor regulatory network for subclusters 24.**

**
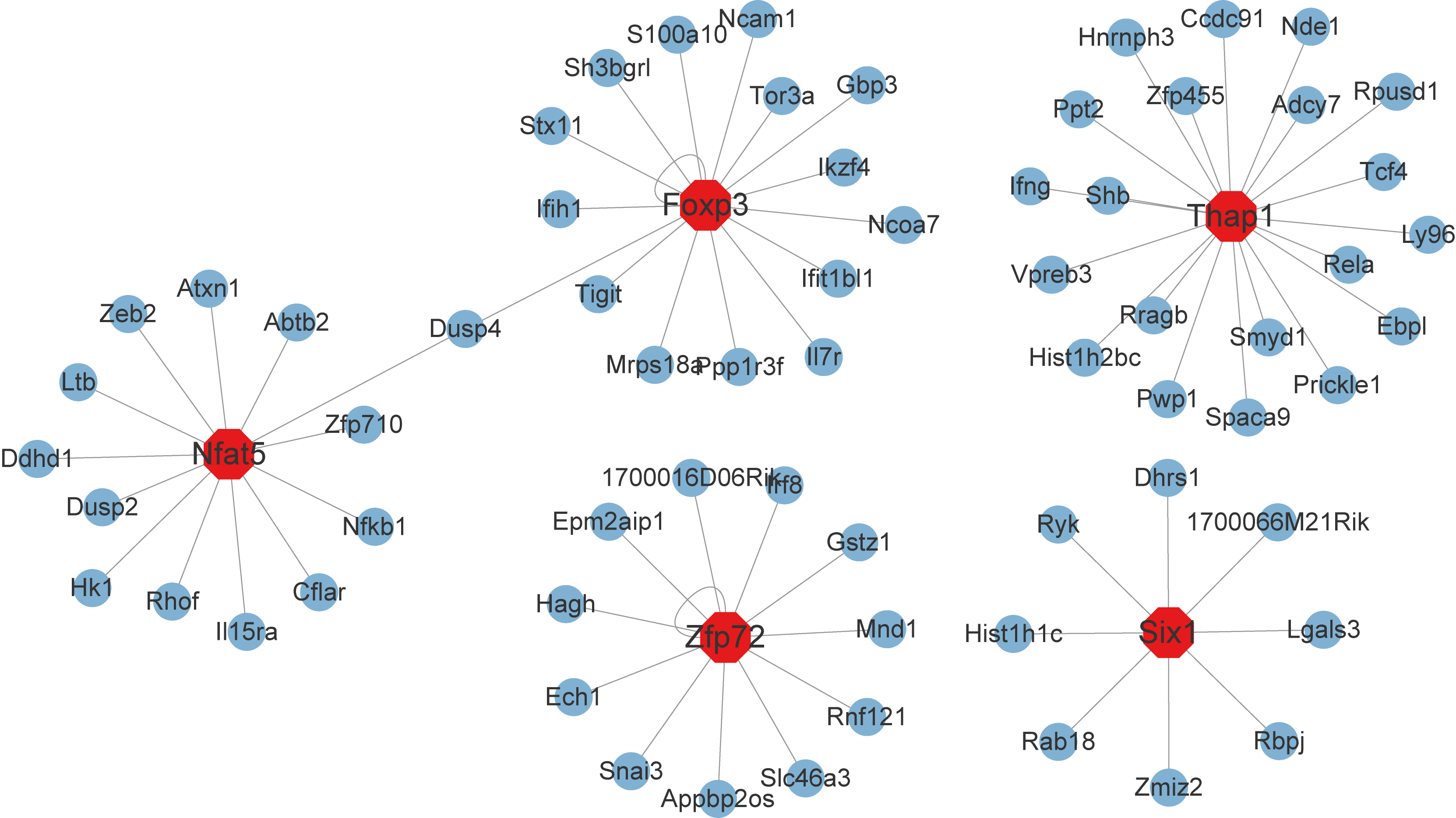
Figure S4. Transcription factor regulatory network for subclusters 27.**

**
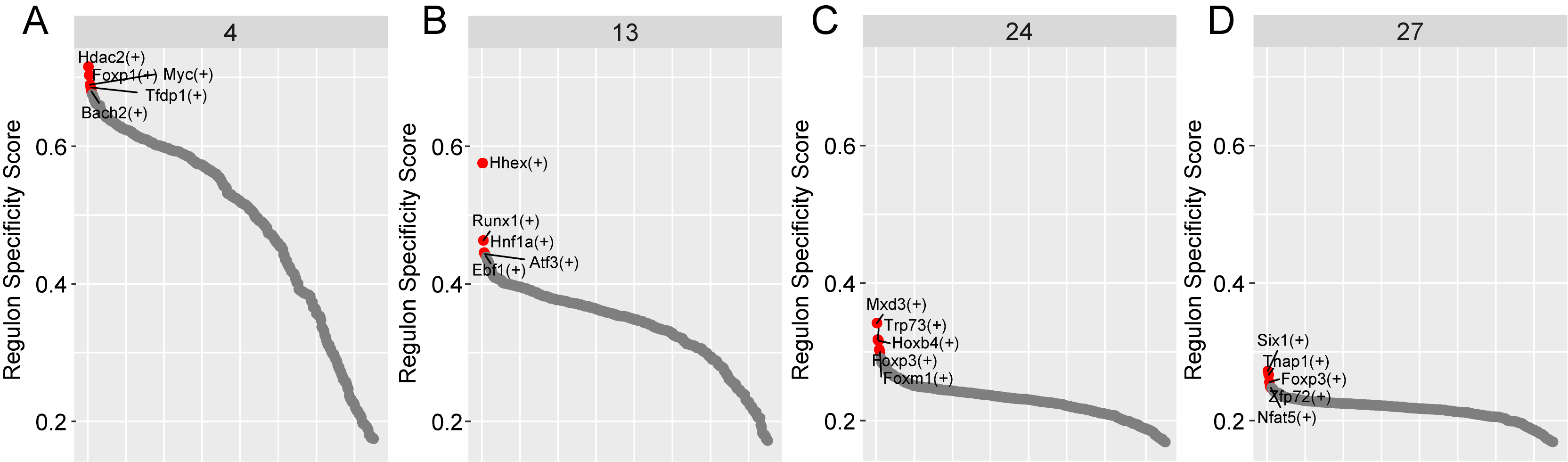
**

**Figure S5. Key transcription factors of subcluster 4, 13, 24, and 27.** (A) Key transcription factors of subcluster 4. (B) Key transcription factors of subcluster 13. (C) Key transcription factors of subcluster 24. (D) Key transcription factors of subcluster 27.

**Table S1. The marker expression pattern of 25 immune cell clusters identified by mass cytometry.**

| Cluster | Cell type | Subtype | Marker expression |
| --- | --- | --- | --- |
| C01 | Neutrophils | / | Gr1^+^Ly6C^hi^Ly6G^+^CD44^hi^TCF7^+^CD11b^hi^ |
| C02 | Neutrophils | / | Gr1^+^Ly6C^hi^Ly6G^+^CD44^hi^TCF7^hi^CD11b^hi^ |
| C03 | MoMF | / | Ki67^+^Ly6C^hi^CD64^hi^CD44^hi^TCF7^hi^CD38^+^CD11b^hi^ |
| C04 | MoMF | / | Ki67^+^MHCII^hi^Ly6C^hi^CD64^hi^CD44^hi^TCF7^hi^CD39^+^CD38^+^CD11b^hi^ |
| C05 | Kupffer cells | / | CD44^hi^F4/80^hi^CD39^+^CD11b^hi^ |
| C06 | Others | / | CCR5^hi^CD64^+^CD44^hi^CCR4^+^NKp46^+^F4/80^hi^TIGIT^+^CD25^+^CD39^hi^CD69^+^PD1^+^KLRG1^+^CD11b^hi^ |
| C07 | cDC | / | MHCII^hi^Ly6C^hi^CD44^hi^CD11c^hi^TCF7^hi^CD39^+^CD38^hi^ |
| C08 | B cells | / | MHCII^hi^CD44^hi^CD38^hi^CD11b^hi^ |
| C09 | B cells | / | Ki67^hi^MHCII^hi^CD38^hi^ |
| C10 | B cells | / | CCR5^+^MHCII^hi^CCR4^+^CD38^hi^ |
| C11 | B cells | / | MHCII^hi^CD44^+^B220^+^CD69^hi^CD38^hi^ |
| C12 | B cells | / | MHCII^hi^B220^+^CD38^hi^ |
| C13 | CD8^+^T | Central memory CD8^+^T | Ly6C^hi^TCF7^+^TCRb^+^CD8a^+^CD127^lo^CD62L^lo^ |
| C14 | CD4^+^T | Effector CD4^+^T | CD44^hi^TCF7^hi^TCRb^+^CD4^+^ |
| C15 | DNT | / | TCF7^hi^Tbet^+^PD1^+^TCRb^+^ |
| C16 | CD4^+^T | Th2 | CCR5^hi^CCR4^+^TCF7^+^CD69^hi^TCRb^+^ |
| C17 | CD8^+^T | Resident CD8^+^T | CD103^+^TCF7^hi^Tbet^+^CD69^hi^TCRb^+^CD8a^+^ |
| C18 | CD4^+^T | Th1 | TCF7^hi^Tbet^+^CD69^hi^TCRb^+^CD4^+^ |
| C19 | NK | / | CD44^hi^CD11c^hi^TCF7^+^NKp46^hi^CD62L^+^Tbet^hi^CD69^+^NK1.1^+^CD38^+^CD11b^hi^ |
| C20 | CD4^+^T | Th1 | CD44^hi^TCF7^+^CD39^hi^Tbet^hi^CD69^hi^TCRb^+^CD38^hi^ |
| C21 | Others | / | CD44^hi^CD11c^hi^TCF7^+^CD39^hi^Tbet^hi^CD69^hi^CD38^hi^ |
| C22 | CD4^+^T | Treg | MHCII^+^CD44^hi^CD103^+^TCF7^hi^TIGIT^hi^CD25^+^CD39^+^CD69^+^TCRb^+^KLRG1^hi^CD38^+^CD4^+^ |
| C23 | gdT | / | CD44^hi^TCF7^hi^Tbet^+^TCRgd^+^CD69^hi^PD1^+^ |
| C24 | Others | / | Ki67^+^MHCII^+^CD44^hi^TCF7^+^ |
| C25 | ILC | / | CD127^+^CD44^hi^CD103^+^TCF7^hi^TCRb^+^CD38^+^ |

**Table S2. The key genes of 31 immune cell subclusters identified by scRNA-seq**

| Cluster | Cell type | Key genes |
| --- | --- | --- |
| 0 | B cells | Ighd, Dmxl1, Bank1, Cd79a |
| 1 | MoMF | Lyz, Chil3, Lpl, Ccr2 |
| 2 | MoMF | Lyz, Slc8a1, Mafb, Pid1 |
| 3 | CD4^+^ T | Tcf7, Cd28, Lef1, Ccr7 |
| 4 | CD8^+^ T | Cd3d, Cd8a, Gzmb, Lef1 |
| 5 | B cells | Cd79b, Agbl1, Ly6d, Cecr2 |
| 6 | B cells | Ighm, Cd79a, Cd79b, Ebf1 |
| 7 | NK cells | Nkg7, Gzma, Prf1, ccl5, ifng, klra4 |
| 8 | Neutrophils | Cxcl2, S100a9, S100a8, Il1b, |
| 9 | Neutrophils | S100a9, S100a8, Retnlg, lcn2 |
| 10 | B cells | ighd, cd83, mdn1, myc |
| 11 | NKT | Nkg7, ifng, icos, zbtb16, furin |
| 12 | MoMF | Lyz, Top2a, Mki67, Fn1 |
| 13 | CD8^+^ T | CD3e, CD8a, Klra7, chn2, ccl5, tox |
| 14 | MoMF | Lyz, Chil3, Lpl, Naaa |
| 15 | CD4^+^ T | Cd3d, cd28, ctla4, tnfrsf4, tox |
| 16 | DPT | Cd3d, Tcf7, lef1, tox, il7r |
| 17 | NKT | CD3dNkg7, Rgs1, gzmc, cd226, tox, ifng |
| 18 | MoMF | cst3, eps8, plbd1, ccr2 |
| 19 | pDC | Siglech, Runx2, rell1, csf2rb2 |
| 20 | Basophils | Prss34, il6, ccl3, ifitm1 |
| 21 | Doublets | Cd3d, Cd8a, ccr7, lef1, ccr7 |
| 22 | MoMF | C1qc, apoe, ccl24, cxcl9, ccl8 |
| 23 | B cells | igha, ighd, ebf1, blk |
| 24 | CD8^+^ T | CD3d, CD8a, top2a, mki67, stmn1 |
| 25 | NK cells | Nkg7, gzma, ifng, klre1 |
| 26 | MoMF | ccl22, fscn1, mreg, cst3, |
| 27 | CD8^+^ T | Cd3d, Cd8a, nkg7, ifng, gzmb |
| 28 | DNT | Cd3d, il7r, il17a, rora, il23r |
| 29 | MoMF | c1qc, fn1, apoe, pf4 |
| 30 | NK cells | Nkg7, gzma, ccl5, klra4 |

**Table S3. Details of the 42 antibodies used for mass cytometry.**

| No | Antibody | Clone ID | Metal Label | Source | Identifier |
| --- | --- | --- | --- | --- | --- |
| 1 | CD45 | 30-F11 | 89Y | BioLegend | Cat# 103102 |
| 2 | CD3ε | 145-2C11 | 115ln | BioLegend | Cat# 100302 |
| 3 | Ki-67 | SolA15 | 139La | eBioscience | Cat# 14-5698-82 |
| 4 | CD195(CCR5) | C34-3448 | 141Pr | BD Biosciences | Cat# 559921 |
| 5 | MHC II(I-A/I-E) | M5/114.15.2 | 142Nd | BioLegend | Cat# 107602 |
| 6 | CD366(Tim-3) | RMT3-23 | 143Nd | BioLegend | Cat# 119702 |
| 7 | CD183(CXCR3) | CXCR3-173 | 144Nd | BioLegend | Cat# 126502 |
| 8 | Gr-1(Ly-6G/Ly-6C) | RB6-8C5 | 145Nd | BioLegend | Cat# 108402 |
| 9 | Ly-6C | HK1.4 | 146Nd | BioLegend | Cat# 128002 |
| 10 | Ly-6G | 1A8 | 147Sm | BioLegend | Cat# 127602 |
| 11 | CD197(CCR7) | 4B12 | 148Nd | BioLegend | Cat# 120101 |
| 12 | CD64(FcγRI) | X54-5/7.1 | 149Sm | BioLegend | Cat# 139302 |
| 13 | CD127(IL-7Rα) | A7R34 | 150Nd | BioLegend | Cat# 135002 |
| 14 | CD44 | IM7 | 151Eu | BioLegend | Cat# 103002 |
| 15 | CD11c | N418 | 152Sm | BioLegend | Cat# 117302 |
| 16 | CD19 | 6D5 | 153Eu | BioLegend | Cat# 115502 |
| 17 | CD194(CCR4) | 2G12 | 154Sm | BioLegend | Cat# 131202 |
| 18 | CD103 | 2E7 | 155Gd | BioLegend | Cat# 121402 |
| 19 | TCF7(TCF1) | 812145 | 156Gd | R&D Systems | Cat# MAB8224 |
| 20 | CD335(NKp46) | 29A1.4 | 157Gd | BioLegend | Cat# 137602 |
| 21 | CD45R(B220) | RA3-6B2 | 158Gd | BioLegend | Cat# 103202 |
| 22 | F4/80 | Cl:A3-1 | 159Tb | Bio-Rad | Cat# MCA497G |
| 23 | CD62L | MEL-14 | 160Gd | BioLegend | Cat# 104402 |
| 24 | TIGIT(VSTM3) | 2190A | 161Dy | R&D Systems | Cat# MAB72671 |
| 25 | FOXP3 | FJK-16s | 162Dy | eBioscience | Cat# 14-5773-82 |
| 26 | CD25 | 3C7 | 163Dy | BioLegend | Cat# 101902 |
| 27 | CD39 | 5F2 | 164Dy | BioLegend | Cat# 135702 |
| 28 | T-bet | 4B10 | 165Ho | BioLegend | Cat# 644802 |
| 29 | TCR γ/δ | GL3 | 166Er | BioLegend | Cat# 118140 |
| 30 | CD206(MMR) | C068C2 | 167Er | BioLegend | Cat# 141702 |
| 31 | CD27 | LG.3A10 | 168Er | BioLegend | Cat# 124202 |
| 32 | CD69 | H1.2F3 | 169Tm | BioLegend | Cat# 104502 |
| 33 | CD161(NK-1.1) | PK136 | 170Er | BioLegend | Cat# 108702 |
| 34 | Gata-3 | TWAJ | 171Yb | eBioscience | Cat# 14-9966-82 |
| 35 | CD279(PD-1) | 29F.1A12 | 172Yb | BioLegend | Cat# 135202 |
| 36 | CD196(CCR6) | 29-2L17 | 173Yb | BioLegend | Cat# 129802 |
| 37 | TCR β chain | H57-597 | 174Yb | BioLegend | Cat# 109202 |
| 38 | KLRG1 | 2F1 | 175Lu | eBioscience | Cat# 16-5893-82 |
| 39 | CD38 | 90 | 176Yb | BioLegend | Cat# 102702 |
| 40 | CD4 | RM4-5 | 197Au | BioLegend | Cat# 100576 |
| 41 | CD8a | 53-6.7 | 198pt | BioLegend | Cat# 100746 |
| 42 | CD11b | M1/70 | 209Bi | BioLegend | Cat# 101202 |
